# The effectiveness of dry heat treatment (fluidotherapy) in patients with distal radius fracture followed conservatively: a single-blinded, randomized controlled clinical study

**DOI:** 10.1590/1806-9282.20250806

**Published:** 2025-12-08

**Authors:** Basak Cigdem-Karacay, Levent Horoz, Ismail Ceylan, Halil Alkan

**Affiliations:** 1 Kirsehir Ahi Evran University, Faculty of Medicine, Department of Physical Medicine and Rehabilitation – Kırşehir, Turkey.; 2 Kirsehir Ahi Evran University, Faculty of Medicine, Department of Orthopedics and Traumatology – Kırşehir, Turkey.; 3 Kırsehir Ahi Evran University, School of Physiotherapy and Rehabilitation – Kırşehir, Turkey.; 4 Mus Alparslan University, Faculty of Health Science, Department of Physiotherapy and Rehabilitation – Muş, Turkey.

**Keywords:** Wrist fractures, Hand, Range of motion, Physical therapy modalities, Edema

## Abstract

**OBJECTIVE:**

The effectiveness of fluidotherapy has not been investigated in patients with distal radius fracturedistal radius fracture.

**AIMS:**

The aim of this study was to investigate the effectiveness of fluidotherapy added to conventional rehabilitation programs on pain, edema, muscle strength, and functionality in conservatively followed distal radius fracture patients.

**METHODS:**

The present randomized controlled, single-blinded study was conducted with 40 distal radius fracture patients who were followed conservatively with a cast. The patients were divided into two groups: the fluidotherapy and conventional rehabilitation groups. In addition to the conventional rehabilitation programs, the patients in the fluidotherapy group received 30 sessions of fluidotherapy for 6 weeks. Rest and activity pain were measured with the Numeric Rating Scale, edema with the Figure 8 method, joint range of motion with goniometry, handgrip strength with a dynamometer, and functionality with the patient-rated wrist evaluation questionnaire. Outcome measures were assessed at baseline, at week 2, and at week 6.

**RESULTS:**

Statistically significant changes were found in the intragroup measurements for all parameters in both groups (p<0.05). In the measurements between the groups, there was a statistical difference in the Numeric Rating Scale rest and range of motion flexion values in the conventional rehabilitation group and in the range of motion pronation parameters in the fluidotherapy group (p<0.05). No difference was found between the groups in the other parameters (p>0.05).

**CONCLUSION:**

Adding fluidotherapy to distal radius fracture rehabilitation had no effect on activity pain, edema, muscle strength, or functionality. Improvement in resting pain among distal radius fracture patients was less with the addition of fluidotherapy. Fluidotherapy was effective only on wrist pronation.

## INTRODUCTION

The prevalence of distal radius fracture (DRF) has been reported as 26–46%, which makes it the most common long bone fracture^1^. In orthopedic follow-up of DRF patients, cases are managed with either surgical methods, such as fixation with a volar plate, or conservative methods, such as casting^2,3^. Early rehabilitation is recommended for both patients who underwent surgery due to DRF and those who were followed conservatively with a cast^4,5^. Early rehabilitation provides more successful results in terms of pain, edema control, muscle strength, and functionality^6,7^.

Electrotherapy methods and physical agents are used for DRF rehabilitation^8,9^. Fluidotherapy, also known as dry heat application, is a type of thermal application. Fluidotherapy units contain spherical pieces of cellulose that circulate in dry air. The aim of fluidotherapy is to transfer heat by convection to the tissue of the extremity placed in the fluidotherapy unit^10^. The effectiveness of fluidotherapy has been investigated in hand osteoarthritis^11^, rheumatoid arthritis^12^, complex regional pain syndrome^13^, and lymphedema developing after breast cancer^14^. However, to the best of the authors’ knowledge, the effectiveness of fluidotherapy has not been investigated in patients with DRF. The current practical application guideline of DRF rehabilitation states that methodologically well-designed studies examine the effect of the fluidotherapy^8^. The aim of this study was to investigate the effectiveness of fluidotherapy added to conventional rehabilitation programs on pain, edema, muscle strength, and functionality in conservatively followed DRF patients.

## METHODS

The present randomized controlled single-blind clinical study was conducted between March 2024 and February 2025 at the Training and Research Hospital.

The study included 40 patients over the age of 18 who were diagnosed with DRFs on two-way radiographs taken after trauma and who were followed up with in a cast because they had no indication for surgical intervention. Cast applications and orthopedic follow-ups of all patients in the study were performed by the same orthopedist (LH). Patients were asked to apply for physical therapy the day after the cast was removed.

Exclusion criteria of the study included polytrauma, history of surgery on the relevant extremity, hemiplegia, contracture, arterial and venous occlusions, lymphatic system disorders, heart and circulatory system disorders, hepatitis, presence of infection, and open wound.

### The application of randomization and blinding

Patients were divided into two groups, namely the fluidotherapy group and the conventional rehabilitation group, by using the computer-assisted randomization method. The physiatrist (BCK) who performed the measurements was unaware of the groups. Different locations were chosen for measurements and interventions so that the investigator would be away from the interventions while performing the measurements. In addition, while the data were transmitted to the statistician, the group names were coded with numbers.

### Outcome measures

Age, gender, weight, height, smoking habit, and dominant hand data of the patients participating in the study were recorded. The patients’ pain intensity at rest and during activity was assessed using the Numerical Rating Scale (NRS). The wrist joint range of motion (ROM) was passively measured using a goniometer. The edema was assessed using the Figure of 8 method with a tape measure^15^. The hand grip strength of the affected extremity was measured using a Jamar^®^ hand dynamometer (Patterson Medical, Warrenville, IL, USA)^16^. Hand grip strength was assessed with the participant seated upright in a chair with armrests. The tested upper extremity was positioned with the shoulder in adduction and the elbow flexed at 90 degrees. Prior to testing, the dynamometer was introduced to the participant. Following a familiarization trial, three consecutive measurements were conducted, and the average of the three trials was used for analysis^17^. The patient-rated wrist evaluation (PRWE) questionnaire was used to assess the functional impairment of the patients^18^. It is recommended that an improvement of more than 11.5 points in PRWE scores be used as the smallest clinically important difference in patients with DRFs^19^. The PRWE questionnaire has cultural adaptation, validity, and reliability^20^. Outcome measures were assessed at baseline (the day after cast removal and before rehabilitation began), at week 2, and at week 6.

### Interventions

Conventional rehabilitation program: All patients in the study received a conventional rehabilitation program. Rehabilitation began with passive ROM stretching and active assisted ROM stretching exercises. After a full ROM was achieved, the strengthening exercises were initiated. The conventional rehabilitation program was applied 5 days a week for 6 weeks, and each session lasted 45 min.

Dry heat treatment (fluidotherapy) program: Patients in the fluidotherapy group received a fluidotherapy program in addition to conventional rehabilitation. For the application of fluidotherapy, the patient was in a sitting position, and the affected extremity was placed in a unit containing finely ground sawdust particles heated with hot air. In this unit, the affected hand was treated under low-pressure conditions with pneumatic pressure (Fizyoflug 2000^®^ Thermotherapy Unit, 230 V/50–60 Hz/2.5 KW, Fizyomed, Turkey). Both heat transfer and tactile massage were aimed to be achieved by immersing the affected hand in the fine sawdust particles. The temperature of the fluidotherapy unit was set at 48°C, and patients received 20 min of treatment under the supervision of a physiotherapist for 6 weeks.

### Sample size

To determine the sample size of the study, G*Power program version 3.1.9.4 (Heinrich-Heine-Universität Düsseldorf, Germany) was used^21^. According to the two-way repeated measures ANOVA with a mixed model test and based on a similar study^14^, the sample power rate was determined as β=80%, type I error rate α=0.05, and effect size d=0.2, resulting in a total of 40 patients (20 patients for each group).

### Statistical analysis

SPSS version 25 software was used for statistical analyses in this study. The conformity of the variables in the study to normal distribution was examined using visual (histogram and probability graphs) and analytical methods (Shapiro-Wilk tests). Descriptive analyses were presented using mean and standard deviation for normally distributed variables. The number and percentage were given for nominal variables. An independent groups t-test was used to compare the values measured in the groups. The Pearson chi-square test was used to compare the groups for categorical variables. Mixed design repeated measures ANOVA was applied to assess the change in the variable, as determined by measurement of the treatment groups over time and group–time interactions. In cases where the sphericity assumption was not met (for 2×3 measurements), Wilks’ lambda test was preferred among multivariate tests. The statistical significance level was accepted as p<0.05.

### Ethical approval

The ethical approval from the University Scientific Research and Publication Ethics Committee was obtained before patient recruitment (Approval Date/Number: 06.02.2024/129944). The study was registered on clinicaltrials.gov.tr after receiving ethics committee approval (NCT06272877). The study was conducted in accordance with the Helsinki Declaration, and written consent forms were obtained from each patient participating in the study. The Consort 2010 guidelines were taken into consideration when designing the study, and it was conducted accordingly^22^.

## RESULTS

Comparisons of the descriptive characteristics of the groups included in the study are given in Table 1. When the descriptive characteristics of the groups included in the study were compared, no statistically significant difference was found (p>0.05).

**Table 1:** Comparison of descriptive characteristics of the groups.

		Fluidotherapy group (n=19)		Conventional rehabilitation group (n=21)		t	p
		X	SD	X	SD		
Age (years)		57	15	58	11	-0.44	0.660
Height (cm)		167	9	164	9	1.17	0.246
Weight (kg)		82	16	74	9	2	0.055
		**n**	**(%)**	**n**	**(%)**	χ^2^	**p-value**
Gender	Female	14	73.7	14	66.7	0.23	0.629
	Male	5	26.3	7	33.3		
Smoking	No	17	89.5	18	85.7	0.12	0.720
	Yes	2	10.5	3	14.3		
Dominant hand	Right	17	89.5	16	76.2	1.21	0.270
	Center	2	10.5	5	23.8		
Operative hand	Right	12	63.2	12	57.1	0.15	0.698
	Center	7	36.8	9	42.9		

t: t-test in independent groups; χ^2^: Chi-square analysis; X: mean; SD: standard deviation.

Time, time–group comparison of the baseline, 2nd week, and 6th week values for NRS (rest, activity), ROM (flexion, extension, supination, and pronation), edema, hand grip strength, and total PRWE measurement data of the study groups are given in Tables 2 and 3. When the changes in NRS (rest and activity), ROM (flexion, extension, supination, and pronation), edema, handgrip strength, and total PRWE measurement data of fluidotherapy and conventional exercise groups were compared over time (intragroup changes), it was found to be statistically significant for all parameters (p<0.05).

**Table 2:** Comparison of the Numeric Rating Scale, range of motion measurement data of the groups at baseline, week 2, and week 6.

			Fluidotherapy group (n=19)		Conventional rehabilitation group (n=21)		Time	Group*time	Pairwise comparisons (week)**	η^2^
								F/p		
			X	SD	X	SD	P			
NRS	Rest	Baseline	2.88	2.32	5.71	1.63	**0.000**	11.36/**0.000**	0–2, 0–6, 2–6	0.230
		Week 2	1.04	1.36	2.10	1.36				
		Week 6	0.25	0.49	0.89	0.53				
	Activity	Baseline	5.49	2.44	6.15	2.02	**0.000**	0.50/0.607	0–2, 0–6, 2–6	0.027
		Week 2	2.16	1.40	2.00	1.21				
		Week 6	0.81	1.10	0.84	0.83				
ROM	Flexion	Baseline	40	13	36	16	**0.000**	13.54/**0.000**	0–2, 0–6, 2–6	0.423
		Week 2	47	10	61	9				
		Week 6	54	10	65	7				
	Extension	Baseline	33	17	36	12	**0.000**	1.51/0.234	0–2, 0–6, 2–6	0.076
		Week 2	46	11	55	8				
		Week 6	51	11	60	6				
	Supination	Baseline	68	22	54	12	**0.000**	2.12/0.134	0–2, 0–6, 2–6	0.103
		Week 2	82	8	78	8				
		Week 6	86	7	82	8				
	Pronation	Baseline	67	17	79	9	**0.000**	3.92/**0.028**	0–2, 0–6, 2–6	0.175
		Week 2	77	9	85	8				
		Week 6	82	9	88	5				

NRS: Numeric Rating Scale, ROM: range of motion; X: mean; SD; standard deviation; η^2^: Effect size.

* Two-way analysis of variance in repeated measures (mixed design repeated measures ANOVA).

** 0: Measurement at baseline, 2: Measurement at week 2; 6: Measurement at week 6. The p-values are written in bold.

**Table 3:** Comparison of the edema, handgrip strength, and total patient-rated wrist evaluation measurement data of the groups at baseline, week 2, and week 6.

		Fluidotherapy group (n=19)		Conventional rehabilitation group (n=21)		Time	Group*time	Pairwise comparisons (week)**	η^2^
							F/p		
		X	SD	X	SD	p			
Edema	Baseline	43.9	3.3	42.5	2.3	**0.000**	1.10/0.342	0–2, 0–6, 2–6	0.056
	Week 2	43.2	3.0	41.7	2.1				
	Week 6	43.0	2.9	41.2	2.0				
Handgrip strength	Baseline	7.06	4.11	11.90	2.73	**0.000**	2.56/0.09	0–2, 0–6, 2–6	0.122
	Week 2	12.16	6.32	15.54	3.49				
	Week 6	16.88	7.03	18.72	3.61				
Total PRWE	Baseline	73.82	15.48	70.35	20.12	**0.000**	0.66/0.520	0–2, 0–6, 2–6	0.035
	Week 2	42.85	15.56	40.15	18.41				
	Week 6	20.72	13.23	21.95	16.26				

Two-way analysis of variance in repeated measures (mixed design repeated measures ANOVA), PRWE: patient-rated wrist evaluation; X: mean; SD: standard deviation; η^2^: effect size.

* Two-way analysis of variance in repeated measures (mixed design repeated measures ANOVA).

** 0: Measurement at baseline, 2: Measurement at week 2; 6: Measurement at week 6. The p-values are written in bold.

In the time–group comparison of NRS (rest and activity), ROM (flexion, extension, supination, and pronation), edema, handgrip strength, and total PRWE measurement data for baseline, week 2, and week 6 values, there was a statistical difference for the parameters such as NRS rest, ROM flexion, and ROM pronation (p<0.05), while no difference was found for the other parameters (p>0.05). While more changes were observed in favor of the exercise group for the variables, NRS rest and ROM flexion, more changes were observed in the fluidotherapy group for the variable ROM pronation (Table 2).

## DISCUSSION

According to the findings of the present study, adding fluidotherapy to the rehabilitation program of conservatively followed DRF patients does not have an additional contribution to edema, functionality, and muscle strength. In addition, fluidotherapy added to conventional treatment showed an improvement in resting pain and joint ROM flexion values that was found to be less than with conventional treatment alone. However, with the addition of fluidotherapy, greater improvement in wrist pronation angle values was found.

Fluidotherapy is a thermal treatment method that has been used for many years in different indications. When used in the therapy of hand osteoarthritis, positive effects have been reported in terms of pain and muscle strength^11^. In patients with rheumatoid arthritis, fluidotherapy was found to be effective on pain, stiffness, and muscle strength, but no effect was found on functionality^12^. Fluidotherapy has been found to be effective in the treatment of neuropathic pain due to complex regional pain syndrome developing in patients after stroke^13^. In this study, adding fluidotherapy to the conventional rehabilitation program after cast removal in patients with DRFs had no effect on activity pain, whereas improvement in resting pain was less with the addition of fluidotherapy. In DRFs, nociceptive pain is observed in the early period, especially at rest, due to direct activation of nociceptors and inflammatory response. It has been reported that a neuropathic component may be added to the pain, especially in operated patients, and it may become chronic. Chronic central pain has been reported to develop in diseases such as hand osteoarthritis and rheumatoid arthritis^23,24^. Similarly, neuropathic pain is detected in complex regional pain syndrome^25^. The authors attributed the low efficacy of pain in patients with DRFs to the different pathophysiological mechanisms of pain and different types of pain.

The edema, which develops especially after cast removal, is still an important problem in patients with DRF^3^. To the authors’ knowledge, no studies have been found that have evaluated the use of fluidotherapy for edema control in orthopedic rehabilitation. However, when fluidotherapy was added to complex decongestive treatment in cases of lymphedema developing after breast surgery, it was found that fluidotherapy was effective in reducing edema^14^. Fluidotherapy has also been reported to be effective in treating edema in patients with post-stroke reflex sympathetic dystrophy^13^. In this study, no effect on edema was found with the addition of fluidotherapy to the conventional rehabilitation program in patients with DRFs. However, it is clear that further research is needed regarding the effectiveness and mechanism of action of fluidotherapy on edema.

This study provides data on early outcomes of DRF rehabilitation, and neuropathic pain was not assessed. Additionally, the short follow-up period of this study can be considered a limitation. However, the study design, the blinding, and the large number of evaluation parameters are the advantages of the present study.

## CONCLUSION

Adding fluidotherapy to the treatment of patients who were followed conservatively due to DRFs had no effect on activity pain, edema, muscle strength, or functionality. The improvement in resting pain of DRF patients was less with the addition of fluidotherapy. Fluidotherapy was only effective in improving wrist pronation.

## Data Availability

The datasets generated and/or analyzed during the current study are available from the corresponding author upon reasonable request.
